# The obesity gene, TMEM18, is of ancient origin, found in majority of neuronal cells in all major brain regions and associated with obesity in severely obese children

**DOI:** 10.1186/1471-2350-11-58

**Published:** 2010-04-09

**Authors:** Markus Sällman Almén, Josefin A Jacobsson, Jafar HA Shaik, Pawel K Olszewski, Jonathan Cedernaes, Johan Alsiö, Smitha Sreedharan, Allen S Levine, Robert Fredriksson, Claude Marcus, Helgi B Schiöth

**Affiliations:** 1 Department of Neuroscience, Functional Pharmacology, Uppsala University, BMC, Uppsala SE 75124, Sweden; 2 Minnesota Obesity Center, Department of Food Science and Nutrition, Saint Paul, MN 55108, USA; 3 Department of Food Science and Nutrition, Saint Paul, MN 55108, USA; 4 Department for Clinical Science, Intervention and Technology, Karolinska Institutet, Division of Pediatrics, National Childhood Obesity Centre, Stockholm, Sweden

## Abstract

**Background:**

TMEM18 is a hypothalamic gene that has recently been linked to obesity and BMI in genome wide association studies. However, the functional properties of TMEM18 are obscure.

**Methods:**

The evolutionary history of TMEM18 was inferred using phylogenetic and bioinformatic methods. The gene's expression profile was investigated with real-time PCR in a panel of rat and mouse tissues and with immunohistochemistry in the mouse brain. Also, gene expression changes were analyzed in three feeding-related mouse models: food deprivation, reward and diet-induced increase in body weight. Finally, we genotyped 502 severely obese and 527 healthy Swedish children for two SNPs near TMEM18 (rs6548238 and rs756131).

**Results:**

TMEM18 was found to be remarkably conserved and present in species that diverged from the human lineage over 1500 million years ago. The TMEM18 gene was widely expressed and detected in the majority of cells in all major brain regions, but was more abundant in neurons than other cell types. We found no significant changes in the hypothalamic and brainstem expression in the feeding-related mouse models. There was a strong association for two SNPs (rs6548238 and rs756131) of the TMEM18 locus with an increased risk for obesity (p = 0.001 and p = 0.002).

**Conclusion:**

We conclude that TMEM18 is involved in both adult and childhood obesity. It is one of the most conserved human obesity genes and it is found in the majority of all brain sites, including the hypothalamus and the brain stem, but it is not regulated in these regions in classical energy homeostatic models.

## Background

Obesity is an increasing global health problem accompanied by several significant health impairments, such as type-2 diabetes and cardiovascular diseases. Although excessive energy intake together with low levels of physical activity contribute to the ongoing obesity epidemic, genetic research has shown that heritage might account for as much as 70% of the population variation in BMI [1]. Genome-wide association (GWA) studies have linked several genomic loci with a high BMI, and several new candidate genes have been identified [2]. Most recently, the FTO gene has been given special attention because of its strong association with obesity in multiple cohorts differing in age [3]. Basic research showed that FTO-deficient mice have less adipose tissue and a lower body mass compared with wild type mice, which supports the biological role proposed for the gene by the GWA analyses [4].

Aside from FTO, GWA studies associated with obesity yet another gene, named TMEM18. TMEM18 was first proposed as an important obesity-related locus by the GIANT consortium [5]. That study strongly associated the nearby SNP, rs6548238, to increased BMI and body weight. Subsequently, these results were replicated in the Icelandic GWA study for three other SNPs located in the close proximity of TMEM18 (rs2867125, rs4854344 and rs7561317), which all showed a strong association to both BMI and body weight [6]. The FTO locus' association to BMI was confirmed therein. Furthermore, the TMEM18 locus' association to BMI has been replicated in two additional studies [7,8] and it has also been associated to the age of menarche [9]. It should be noted that while the risk contribution of the TMEM18 locus to obesity in adults has been examined (6), no attempt to determine such association has been made in obese children. However, one previous study has associated the locus with BMI in children [8].

The TMEM18 gene codes for a poorly characterized transmembrane protein. One study indicated that this protein is located in the nuclear envelope in neural stem cells [10]. TMEM18 may be involved in cell migration as overexpression of the protein increases the migration of neural stem cells towards glioma in the rat brain. A preliminary expression profiling of TMEM18, that accompanied the first GWA study, suggested that it is ubiquitously expressed, but with certain differences between tissues [5]. In line with the proposed role in the regulation of body weight, TMEM18 was shown to be expressed in the brain, including the hypothalamus, the region responsible for the control of energy homeostasis. Only few other brain areas were investigated in that project.

The current project provides the first detailed characterization of the human TMEM18 protein's sequence features, evolutionary history, gene expression profile in the rat and mouse, distribution in the mouse brain, and gene expression regulation in several murine feeding/body weight models. We also investigated the risk of obesity for variants upstream of the TMEM18 locus in the cohort of severely obese children and healthy controls, and performed the first examination of the locus association to several obesity-related traits.

## Methods

### Evolutionary and sequence analysis

#### Retrieval of protein sequences

The proteomes of eleven different organisms representing different lineages of eukaryotes, with the focus on the metazoans and vertebrates, were gathered from different sources as follows. All protein sequences for *Homo sapiens, Mus musculus, Gallus gallus, Xenopus tropicalis, Caenorhabditis elegans *and *Drosophila melanogaster *were downloaded from Ensembl 53 [11]; *Branciostoma floridae *[12],*Trichoplax adhaerens *[13] and *Thalassiosira pseudonana *[14] were downloaded from the Joint Genome Institute's website, *Saccharomyces cerevisae *was downloaded from the *Saccharomyces *genome database [15], *Arabidopsis thaliana *from The Arabidopsis Information Resource (TAIR) [16] and all bacterial proteomes were downloaded from NCBI's ftp-site [17].

#### Mining for TMEM18

The human TMEM18 sequence was collected from UniProt [18] and used to query the OMA database [19] (release September 2008). The OMA is a database which automatically groups protein sequences predicted to be orthologous. To allow identification of members of the TMEM18 family that are not orthologous to the human gene, we used the following procedure. Sequences that belong to the orthologous group of human TMEM18 were downloaded from the database and aligned using mafft-einsi [20] with default parameters. The multiple sequence alignment (MSA) was viewed and edited in Jalview [21]. A hidden-markov model (HMM) was created and calibrated from the MSA using the HMMER [22] package with default parameters. The HMM was applied on the listed proteomes using the hmmpfam program of the HMMER package with the default parameters. The results were evaluated for false-positives and hits with an E-value below 10^-6 ^were considered as significant. The protein and nucleotide sequences for the hits were collected and redundancy was manually removed so that only one protein/transcript was kept for each gene.

#### Multiple sequence alignments and Phylogenetic analysis

The protein sequences identified in the mining procedure were aligned using mafft-einsi with default parameters. The resulting MSA was viewed, analyzed and edited in Jalview. The protein sequences of the MSA were reversely translated to the corresponding mRNA transcripts by using RevTrans [23] with the default parameters and the retrieved nucleotide MSA was used for the phylogenetic analysis. The phylogenetic inference was performed with the maximum likelihood method using RAxML (v. 7.0.4) [24] according to the "Hard & Slow Way" described in the program's manual, the EasyRAx script found at the program's website was utilized to execute the analysis. In brief, the best known maximum likelihood tree was found in 100 maximum parsimony trees, 1000 non-parametric bootstraps were carried out and frequencies were written on the best known maximum likelihood tree. The final tree was drawn using Mesquite (v 2.6) and edited in Inkscape (v. 0.46), using the *T. pseudonana *sequence as the outgroup. Pairwise sequence alignments were made using EMBOSS' [25] implementation of the Needleman-Wunsch [26] algorithm with the default parameters.

#### Sequence analysis of the human TMEM18 protein

Poly Phobius [27] (v. 1.04) was used to predict transmembrane topology for the human TMEM18 sequence. The protein MSA made for the evolutionary analysis was used as input to increase accuracy of the prediction. Glycosylation sites of type O-β-GlcNAc, which occurs in the nucleus, was predicted using the YinOYang [28] (v. 1.2) server with the default parameters. Phosphorylation sites were predicted with the NetPhos [29] (v. 2.0) server using the default parameters. The prediction of nuclear localization signals (NLS) was performed with PredictNLS and the NLSdb database [30]. Finally, the UniProt and Ensembl websites were consulted for additional annotation.

### TMEM18 tissue expression profiling with quantitative real-time PCR

The TMEM18 mRNA expression was detected in tissue panels from both the mouse and the rat. Animal handling and tissue panel preparation was performed as previously described in Haitina et al. together with details of the real-time PCR analysis [31]. The primers used can be found in Additional file 1. All animal experiments has been approved by "Uppsala's Ethical Committee on Animal Experiments" (reference number: C285/5 and C84/8) and follows international guidelines.

### TMEM18-like immunoreactivity in the murine brain

C57Bl6/J adult male mice were housed in the controlled environment (21°C, 12:12 LD cycle), with *ad libitum *access to chow and water. An intraperitonial injection of mixture of Dormitor (70 μg/g b. wt., Orion, Finland) and Ketalar (7 μg/g b. wt., Pfizer, Sweden) was used to anesthetize the mice. Phosphate buffered saline (PBS) followed by 4% paraformaldehyde were used to perform transcardial perfusion through the left ventricle. The fixed brain was excised and incubated overnight in 4% paraformaldehyde; subsequently the tissue was dehydrated and infiltrated with paraffin (Tissue Tek vacuum infiltration processor; Miles Scientific, Elkhart, IN). Sectioning (7 μm) of the paraffin-embedded brain was carried out on the Microm microtome onto superfrost slides (Menzel-Gläser, Braunschweig, Germany).

#### Immunoflourescence

Sections were deparaffinized in X-tra solve (Medite Histotechnic, Burgdorf, Germany) and rehydrated with a series of washes in descending concentrations of ethanol followed by autoclaved MilliQ water. Antigen retrieval was performed by boiling the sections in 0.01 M citric acid (pH 6.0) for 10 minutes. Slides were cooled and rinsed with 1× PBS pH 7.4. They were transferred to the humidity (PBS) chamber. Primary antibodies: rabbit anti-TMEM18, (ProSci, Poway, CA) and mouse anti-NeuN (Neuronal Nuclei, Millipore, Temecula CA, USA) diluted 1:400 in PBS+0.3% Triton X100 (Sigma-Aldrich, USA) were added onto the slides and incubation was carried out overnight at 4°C. The slides were then washed with PBS and incubated in the secondary antibodies: donkey-anti-rabbit 594 (1:200; Alexa Fluor, Invitrogen, USA) and goat-anti-mouse 488 (1:400; Alexa Fluor, Invitrogen, USA), respectively in PBS+0.3% Triton X100 in the dark chamber for 4 hours. The slides were washed with PBS and stained with DAPI. They were coverslipped using DTG (2.5% DABCO (Sigma), 50 mM Tris-HCl pH 8.0, 90% glycerol). Sections were analyzed under the Zeiss XBO75 microscope.

#### Analysis of results

Pictures of the cortex, amygdala, hypothalamus, thalamus and hippocampus were taken with the Zeiss AxioCam HRm under 20× magnification. Photomicrographs show a random portion of each region of interest. The images were analyzed and cells counted through appropriate filters using ImageJ 1.41o software (National Institute of Health, USA). A total of 5 pictures from the cortex, 4 from the amygdala, and 3 each from the hypothalamus, thalamus and hippocampus were analyzed. The total number of all cells, NeuN cells and TMEM18-positive cells as well as the number of TMEM18 expressing neurons and non-neurons were counted separately. Percentages of neurons expressing TMEM18, non-neurons expressing TMEM18, and all cells combined expressing TMEM18 were calculated.

### Feeding experiments

Male C57BL/6J mice (Scanbur, Sweden), housed individually or in groups of two (Exp. 1, 2 and 3) in macrolon cages, under LD 12:12 (lights on at 0700), weighed ca. 28 g at the beginning of the experiment. Tap water and chow (Lactamin, Sweden) were available ad libitum unless specified otherwise. Procedures described herein were approved by the Uppsala Animal Welfare Committee and followed the EU and Swedish guidelines.

#### Experiment 1. Hypothalamic and brainstem TMEM18 mRNA levels following 16-h and 24-h of food deprivation

Chow was removed before the onset of the dark phase and mice (n = 8) were decapitated between 1000 and 1100 on the next day. Control mice (n = 8) had ad libitum access to food.

#### Experiment 2. Hypothalamic and brainstem TMEM18 mRNA levels following 48-h consumption of palatable sucrose or Intralipid

Mice gained access to a bottle containing 10% sucrose or 4.1% Intralipid (Fresenius, Sweden) for 48 h; control animals had access to chow only (n = 8/group). Intralipid, a palatable lipid emulsion, has been used in experiments utilizing liquid diets [32,33]. Sucrose and Intralipid were isocaloric (0.4 kcal/g, 1 kcal = 4.2 kJ); energy density of chow was 3.6 kcal/g. The solutions were similar in palatability: each mouse ingested on average 8.1 kcal of Intralipid and 7.4 kcal of sucrose per day. Total energy intake per animal was 10.3 kcal in the chow group, 14.1 kcal in the sucrose group and 12.9 kcal in the Intralipid group. Mice were decapitated after 48 h (1100 - 1200).

#### Experiment 3. Hypothalamic and brainstem TMEM18 mRNA levels upon increased body weight

Mice received 10% sucrose in addition to chow for 3 weeks; controls had chow only (n = 8/group). The initial body weights did not differ significantly between the two groups (controls: 27.6 ± 0.3 g; sucrose: 27.8 ± 0.5 g). At endpoint, sucrose-fed mice weighed 32.1 ± 0.4 g, whereas the control animals weighed 29.5 ± 0.6 g (P < 0.05; t-test). Energy intake was similar to that described for the corresponding groups in Experiment 1. Mice were decapitated between 1100 and 1200.

### Analysis of genetic association to obesity and related traits

#### Phenotype characterization

Birth weights and lengths were obtained from growth charts. Actual body weights and lengths were measured to the nearest 0.1 kg and 1 cm, respectively. BMI standard deviation score (BMI SDS) was calculated from weight, height, standardized for age and gender [34]. For the obese subjects, levels of plasma glucose, serum insulin, serum triglycerides and cholesterol were analysed from blood samples drawn after 16-h overnight fasting as previously described [35].

#### Subjects

We genotyped 1027 children and adolescents comprising two study groups as described earlier [35]. Briefly, a case group of 502 obese children (262 girls and 240 boys) was enrolled at the National Childhood Obesity Centre at Karolinska University Hospital, Huddinge, Sweden. The obese subjects were between 6 and 20 years old and mean age was 12.6 ± 3.3 years. BMI SDS ranged from 2.7 to 11.1 and mean BMI SDS was 6.2 ± 1.4 2) The control group with 525 healthy Swedish adolescents (268 girls and 257 boys) was recruited from 17 upper secondary schools in the Stockholm area. The subjects in the control group were between 15 and 20 years old and mean age was 17.1 ± 0.8 years. BMI SDS ranged from -1.9 to 2.4 and mean BMI SDS was 0.2 ± 0.8. Subjects with overweight/obesity or chronic diseases were excluded from the control group and seven children with type-2 diabetes were excluded from the obese group. The study was approved by the Regional Committee of Ethics, Stockholm, (reference number KI702/03) and follows the Helsinki declaration and international guidelines. All participants or their legal guardians gave their written informed consent of inclusion in the study and publication of the results.

#### Statistical analysis

In order to test for deviation from Hardy-Weinberg equilibrium, the Pearson's χ^2^-test (1 d.f) was applied. Genotype and allele frequencies were calculated and logistic regression was used to calculate the odds ratio (OR) with a 95% confidence interval (CI) assuming an additive model. Association with obesity was determined by comparing subjects with normal weight and obesity. Associations between genotypes and phenotypes were analyzed with linear regression, assuming an additive model. Quantitative skewed variables were log-transformed before analysis. Covariates such as birth weight, birth length, weight, length, gender, BMI SDS and age were tested for dependence on the response variables and included in the model if significant. All the analysis was performed using PLINK [36].

#### Genotyping

Genomic DNA was extracted from peripheral blood using QiaGen Maxiprep kit (Qiagen, Hilden, Germany). Genotyping was performed with pre-designed Taqman single-nucleotide polymorphism genotyping assay (Applied Biosystems, Foster City, USA) and an ABI7900 genetic analyser with SDS 2.2 software.

## Results

### Sequence analysis of the human TMEM18 protein

**Figure 1:**
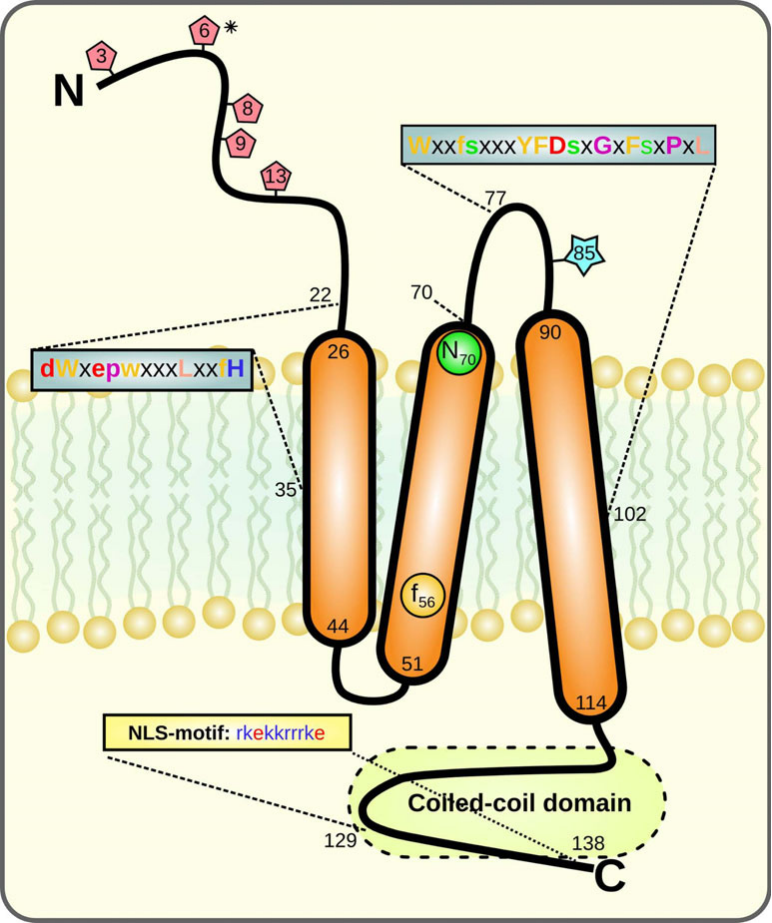
**Schematic image of the human TMEM18 protein's features**. TMEM18 has three transmembrane α-helices and the N-terminal and second loop probably face the inside of the nucleus, whereas the first loop and the C-terminal are oriented towards the cytoplasm, according to predictions by Phobius. The N-terminal contains five predicted O-β-GlcNAc glycosylation sites (red pentagons) and the second loop contains one phosphorylation site (blue star). Also, one of the glycosylation sites (*) is potentially targeted by phosphorylation. The C-terminal is annotated to contain a Coiled-coiled domain and an NLS-motif. The protein is highly conserved: blue boxes represents well conserved motifs, capital letters represent amino acids that are completely conserved in all investigated organisms and lower cases represent those conserved in 90%. Circles represent single conserved residues. The circles and amino acid letters are colored according to Zappo color scheme in Jalview. All numbers represent amino acid positions, starting at the N-terminal.

A comprehensive picture of the human TMEM18 protein can be found in Figure 1. The human TMEM18 gene is located on chromosome 2. It is 9466 bp-long, including introns, and it has seven protein coding exons. The gene has three transcripts of which the longest one is translated into a 140 amino acid-long protein, that were predicted to have three transmembrane α-helices. The N-terminus of the protein, which probably faces the inside of the nuclear envelope, was predicted to have five O-β-GlcNAc glycosylation sites, and the second loop to have one phosphorylation site. The protein has a nuclear localization signal at the C-terminal, which according to NLSdb, is identical to, among others, human homeobox proteins and it targets the protein for transport to the nucleus. A coiled-coil domain was also found in the same part of the protein.

### Evolutionary investigation

**Figure 2:**
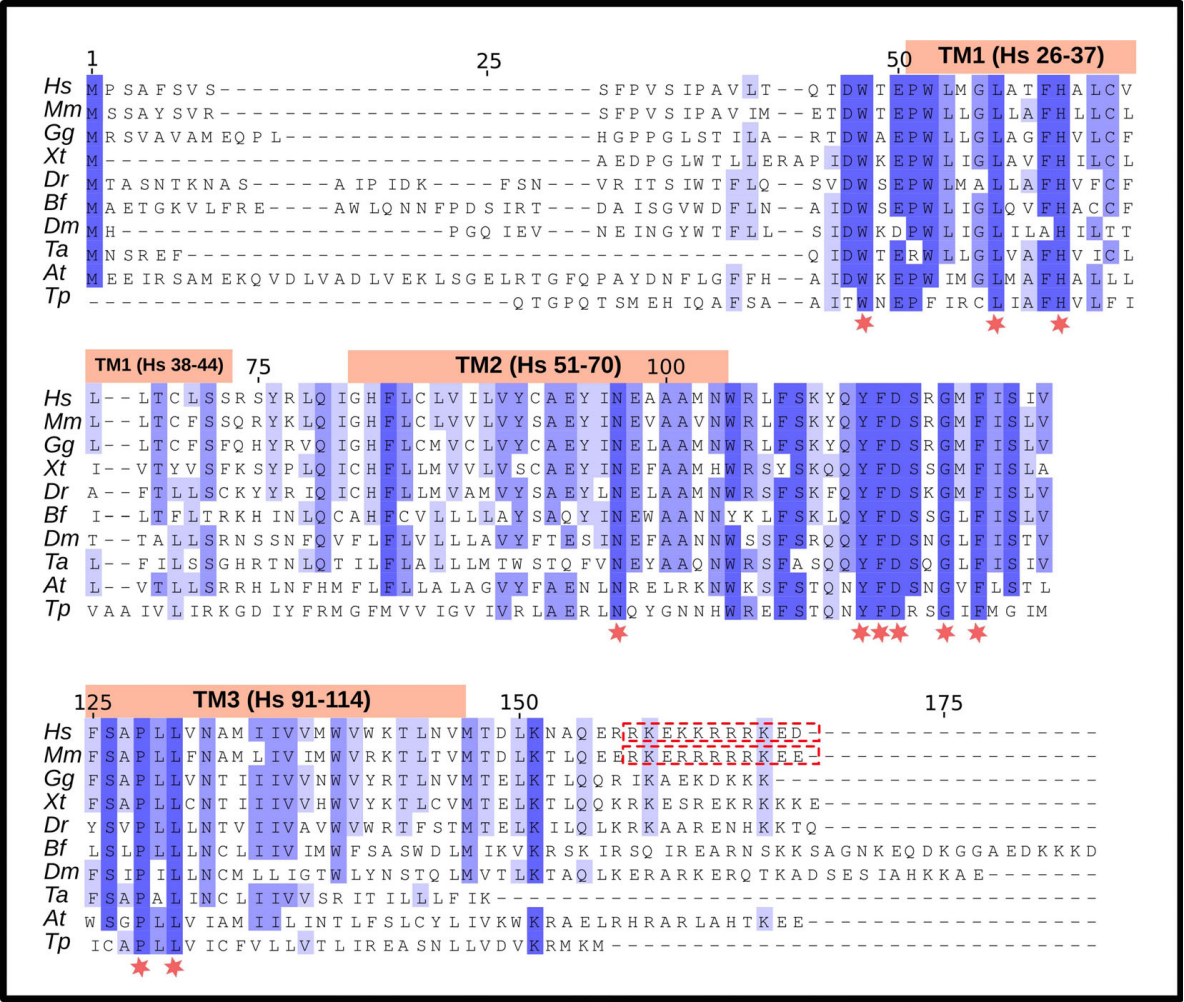
**Multiple sequence alignment of the TMEM18 family**. The protein sequences were aligned using mafft-einsi with the default parameters and viewed and edited with Jalview. The column color intensity corresponds to the degree of conservation and completely conserved residues are marked with a red star. The predicted transmembrane helices of the human TMEM18 protein are represented by pink boxes and the predicted nuclear localization signals for the mouse and human are marked with red boxes in the sequence.

We searched for homologues in a wide range of genomes in all kingdoms of life and we present herein the specific mining of eleven genomes for genes homologous to TMEM18. We found that all genomes contained one single positive hit for the TMEM18 family, except for *S. cerevisae *and *C. elegans *where no homologous genes were identified. The non-redundant proteins sequences used that were received in the mining is available in Additional file 2. We investigated this further by mining the NCBI's protein database nr using protein PSI-BLAST with the human TMEM18 protein sequence as query and with the default parameters. We found no sequences from the fungi or *pseudoocoelomata *(where *C. elegans *belongs) lineages in these searches, suggesting that these taxonomic groups have lost the TMEM18 gene. The multiple sequence alignment (Figure 2) showed that the TMEM18 sequence is well conserved, eleven residues were identical in all the investigated organisms and another 30 were identical with 80% of the sequences. The average pair wise sequence identity (similarity) was 37% (82%) for all species and 41% (88%) within vertebrates. Thus, the TMEM18 family has been highly conserved during the evolutionary history of eukaryotes.

**Figure 3:**
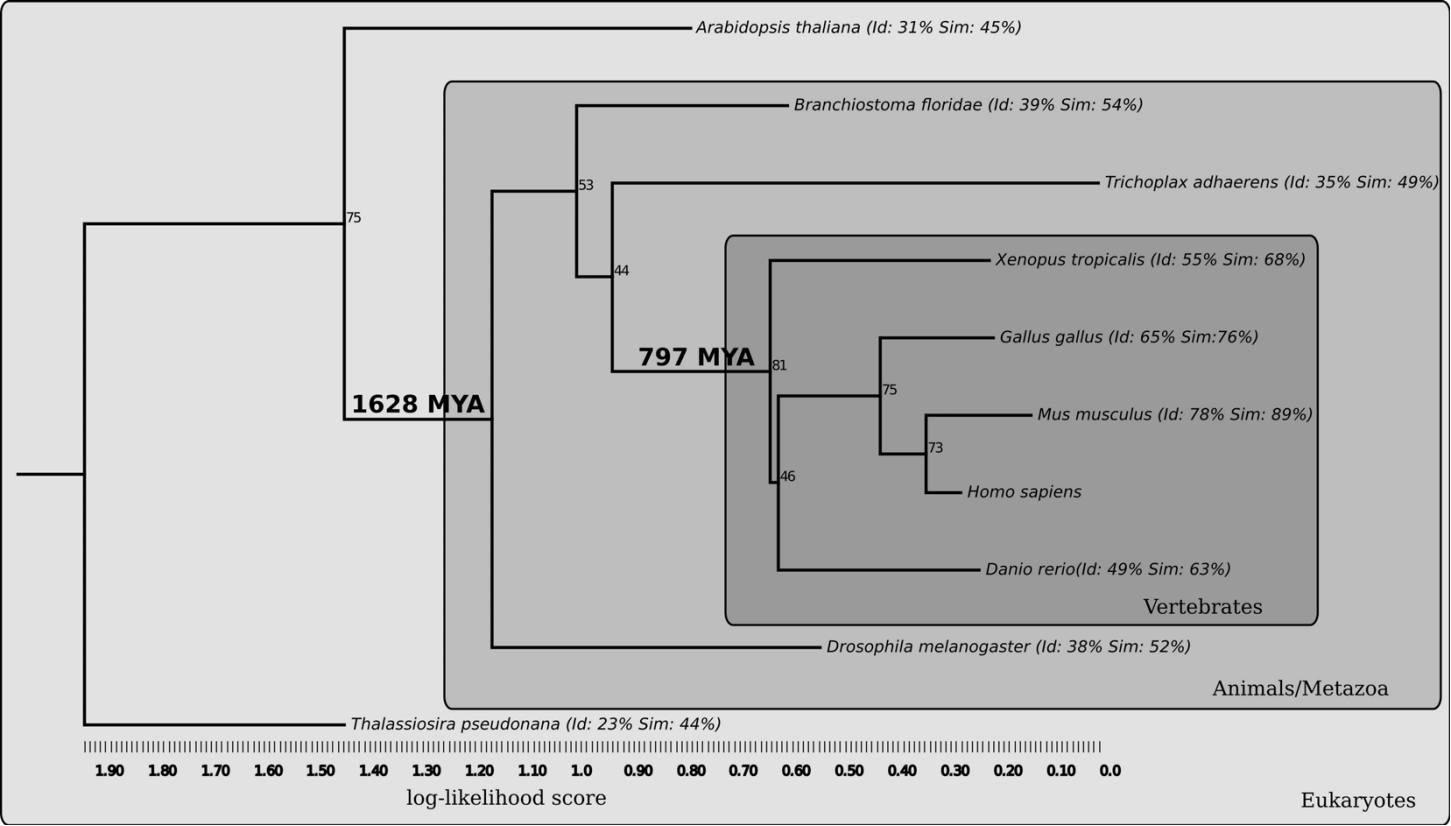
**Phylogenetic tree of the TMEM18 family**. The phylogenetic tree was calculated from a nucleotide multiple sequence alignment with a maximum-likelihood approach using the RAxML program. The tree was bootstrapped 1000 times and branch lengths correspond to log-likelihood scores (see scale). The sequence similarities and identities (percentage of alignment length) for each protein are towards the human TMEM18 protein and were calculated from global alignments produced with the EMBOSS implementation of the Needleman-Wunsch algorithm. The time points since the divergence of animals and plants/*T. pseudonana *(1625 MYA) and since the radiation of vertebrates (797 MYA) were taken from the TimeTree resource.

We created a phylogenetic tree for the TMEM18 family (Figure 3). *T. pseudonana *was used as the outgroup and, as expected; the plant *A. thaliana *was found to be basal of the metazoan group. Within the metazoan group, the order of invertebrates was not as anticipated, since *T. adhaerens *should be most basal, followed by *D. melanogaster *and *B. floridae*. However, the supporting bootstrap values were not strong for this part of the topology, which may reflect high conservation of the sequences. The vertebrates formed a clear cluster with an expected topology, apart from the reversal of the *X. tropicalis *and *D. rerio *positions, but here the bootstrap values were low.

### Expression profiling

**Figure 4:**
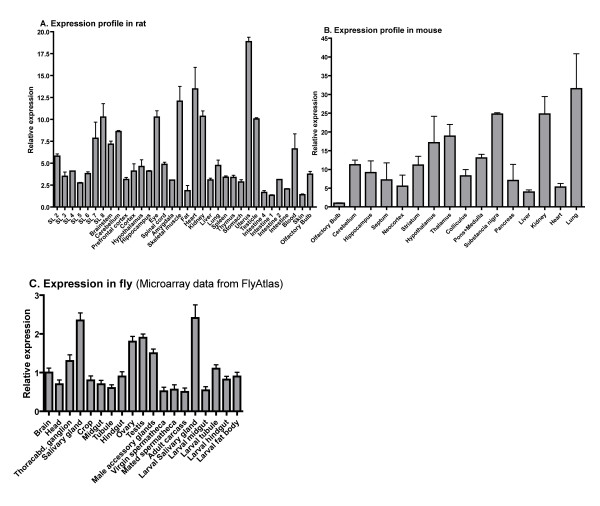
**TMEM18 tissue distribution in rat, mouse and fly**. Relative mRNA expression of the TMEM18 gene was investigated in three species. Means are relative to the tissue with the lowest expression (set to 1) in the rat and mouse. The expression was measured with real-time PCR for **A**. Rat and **B**. Mouse, whereas the expression of TMEM18 in **C**. fly was received from the FlyAtlas microarray database, the expression is relative to the whole fly. All error bars represent SEM.

We investigated the TMEM18's mRNA expression pattern in a wide range of rat and mouse tissues using real-time PCR. We found that TMEM18 was widely expressed, but varied in expression level between different tissues and was abundant in feeding and body weight regulatory brain regions such as the hypothalamus and brainstem, although higher in other tissues (Figure 4). As a comparison, we downloaded microarray expression data for the *D. melanogaster *TMEM18 from FlyAtlas [37], which confirmed the wide expression pattern found in the rat and mouse (Figure 4)

### Immunohistochemical detection of TMEM18 in the mouse brain

**Figure 5:**
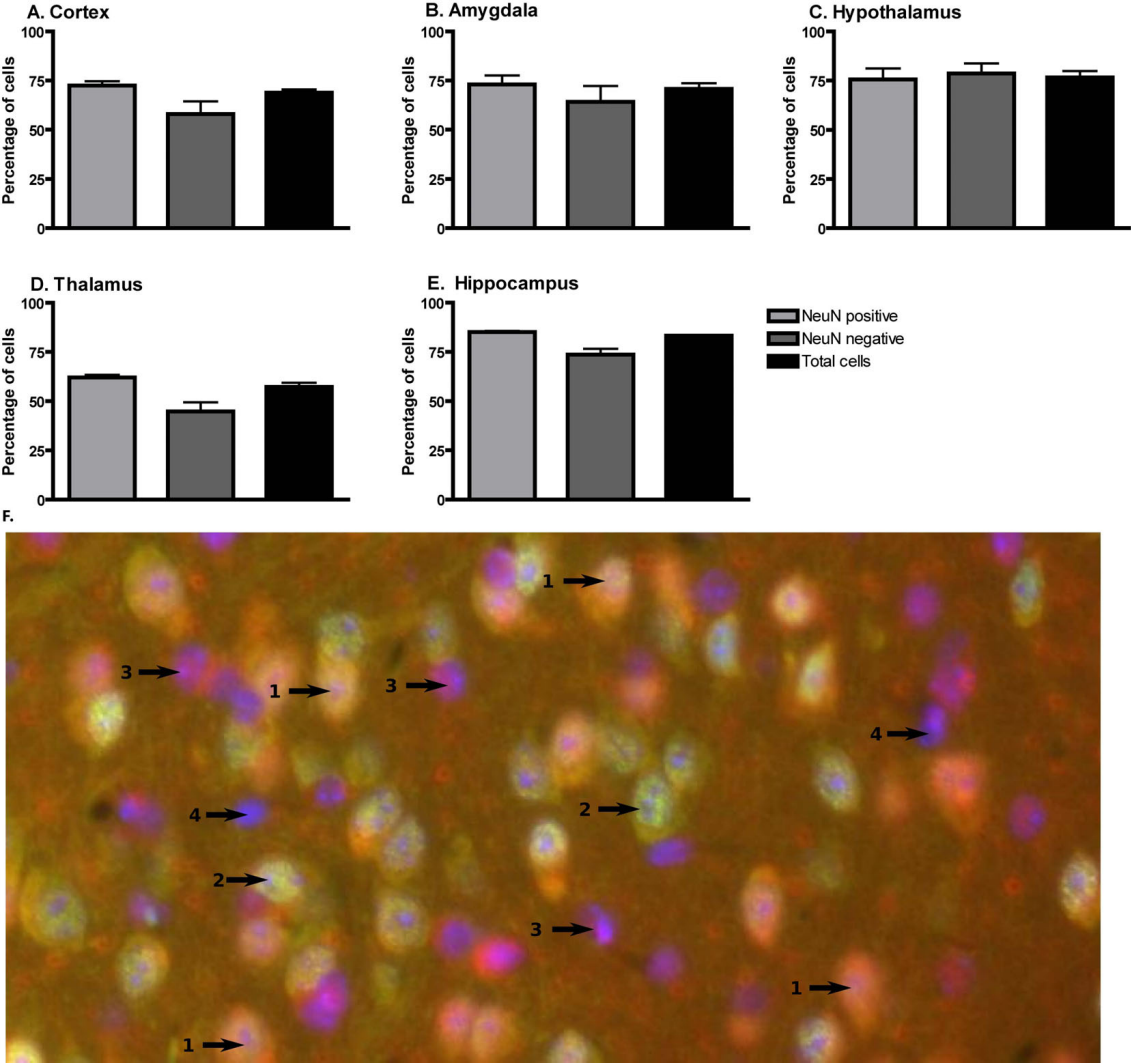
**The TMEM18 protein distribution in different cells in the mouse brain**. Averaged percentages of different cell types positive for TMEM18 in five regions of the mouse brain: **A**. Cortex, **B**. Amygdala **C**. Hypothalamus **D**. Thalamus and **E**. Hippocampus. Sections were stained with DAPI (blue) as a cellular marker, NeuN antibody (green) for neurons and TMEM18 antibody (red). **F**. cell types in the hypothalamus are marked with arrows and numbered as follows: **1**. TMEM18-positive neurons, **2**. TMEM18-negative neurons, **3**. TMEM18-positive non-neurons and **4**. TMEM18-negative non-neurons.

We stained a large number of sections from the mouse brain. No region was devoid of TMEM18 immunoreactivity and the TMEM18 protein showed a widespread distribution throughout the mouse brain. We also performed co-labeling of TMEM18 with the cellular marker, DAPI, and a neuronal marker, NeuN (Figure 5) and in total 7276 cells from the cortex (1904 cells), amygdala (1505 cells), hypothalamus (1314 cells), thalamus (1033 cells) and hippocampus (1520 cells) were counted. The results revealed that TMEM18 was present in 71% of all cell types, 73% of NeuN-positive cells and 63% of NeuN-negative cells (Figure 5).

### Central expression of TMEM18 in mice differing in feeding or body weight profiles

**Figure 6:**
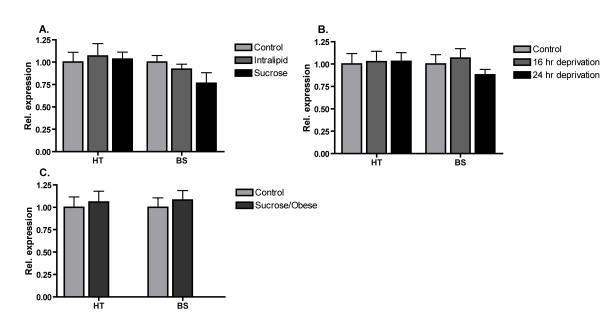
**TMEM18 hypothalamic and brainstem mRNA expression in three feeding-related mouse models**. mRNA expression relative to the control group (error bars represent SEM) in the hypothalamus (HT) and brainstem (BS) for: **A**. 48-h consumption of palatable sucrose or Intralipd, **B**. 16- and 24-h food deprivation and **C**. Increased body weight after 3-week exposure to sucrose. No significant changes in TMEM18 mRNA expression were found using a one-way ANOVA in any of the experiments.

Hypothalamic and brainstem expression of TMEM18 was unchanged in all of the feeding paradigms utilized herein, including 16- and 24-h energy deprivation, as well as short- (48 h) and long-term (3 weeks long; leading to increased body weight) exposure to palatable tastants that differ in macronutrient composition (see Figure 6).

### Analysis of genetic association to obesity and related traits

None of the two SNPs analyzed caused deviation from Hardy-Weinberg equilibrium (Table 1) and the variants were almost in complete linkage disequilibrium, r^2 ^= 0.95. Table 1 shows obesity risk estimates (odds ratios; OR) for the two genotyped variants located 23 kbp upstream TMEM18, rs6548238 and rs7561317. Both SNPs were significantly (p = 0.002 and p = 0.001) associated with obesity in directions consistent with prior reports of association with BMI (OR = 1.450 and OR = 1.507) [5-7]. Table 2 summarizes the analyzed phenotypes in the normal weight and obese children and adolescents for the two SNPs. No significant association was found after adjustment for multiple testing. There was, however, a clear trend for BMI SDS among the obese children: the homozygote carriers of the major allele had 0.5 SD higher BMI SDS levels compared to the homozygote carriers of the minor allele (p = 0.073 and p = 0.038). This trend was also observed among the obese children for a shorter birth length for carriers of the major allele for both SNPs (p = 0.048 and p = 0.032).

**Table 1:** Association study of *TMEM18 *rs6548238 and rs7561317 variants with obesity

	n	Genotype, n (%)			MAF, %	OR (95% CI)	P	HWE
						
		11	12	22				
**rs6548238**		**CC**	**CT**	**TT**				
Normal weight	523	356 (68)	148 (28)	19 (4)	18			0.461
Obese	500	378 (76)	115 (23)	7 (1)	13	1.450 (1.137-1.850)	0.002	0.586
								
**rs7561317**		**GG**	**GA**	**AA**				
Normal weight	523	348 (67)	156 (30)	19 (4)	19		0.001	0.770
Obese	499	375 (75)	117 (23)	7 (1)	13	1.507 (1.184-1.918)		0.530

Data are number of subjects in each group and number of subjects for each genotype (% in each group). Minor allele frequency (MAF) for each group is given as percentage. Odds ratio (OR) with a 95% confidence interval (CI) was calculated assuming an additive model. Association with obesity was determined comparing subjects with normal weight and with obesity. P indicates p-values adjusted for sex. HWE indicates p-values for deviation from Hardy-Weinberg Equilibrium.

**Table 2:** Anthrometric characteristics in obese and normal-weight children and adolescents stratified according to *TMEM18 *rs6548238 and rs7561317 genotype.

rs6548238	CC	CT	TT	P
**Normal-weight, n = 523**				
Age (years)	17.1 ± 0.9	16.9 ± 0.8	17.0 ± 0.5	0.126
Weight (kg)	63.5 ± 9.9	63.5 ± 10.1	65.2 ± 9.6	0.247
Length (m)	1.73 ± 0.08	1.73 ± 0.09	1.73 ± 0.09	0.651
BMI SDS	0.139 ± 0.565	0.184 ± 0.630	0.158 ± 0.375	0.921
**Obese, n = 500**				
Age (years)	12.2 ± 3.3	12.6 ± 2.9	13.2 ± 2.5	0.203
Birth length (cm)	50.0 ± 2.9	50.4 ± 2.5	52.1 ± 1.6	0.048
Birth weight (g)	3465 ± 721	3627 ± 641	3879 ± 225	0.077
Length (m)	1.66 ± 0.14	1.58 ± 0.16	1.58 ± 0.18	0.674
Weight (kg)	109.6 ± 31.5	90.4 ± 28.5	91.5 ± 29.1	0.920
BMI SDS	6.3 ± 0.9	5.5 ± 1.6	5.8 ± 1.5	0.073
P-Glucose (mmol/l)	4.5 ± 0.7	4.3 ± 0.6	4.4 ± 0.9	0.512
fS-Insulin (mmol/l)	118.2 ± 71.3	113.8 ± 74.5	92.6 ± 44.3	0.503
Triglycerides (mmol/l)	0.82 ± 0.81	0.82 ± 0.97	0.57 ± 0.53	0.944
Cholesterol (mmol/l)	3.73 ± 0.85	3.84 ± 0.86	3.43 ± 0.53	0.482
				

**rs7561317**	**GG**	**GA**	**AA**	**P**

**Normal-weight, n = 523**				
Age (years)	17.1 ± 0.9	16.9 ± 0.8	17.1 ± 0.5	0.175
Weight (kg)	63.4 ± 9.9	63.6 ± 9.9	65.2 ± 9.6	0.335
Length (m)	1.72 ± 0.09	1.73 ± 0.09	1.73 ± 0.09	0.708
BMI SDS	0.139 ± 0.567	0.181 ± 0.629	0.158 ± 0.374	0.780
**Obese, n = 500**				
Age (years)	12.2 ± 3.3	12.6 ± 2.9	13.3 ± 2.5	0.191
Birth length (cm)	49.9 ± 2.9	50.4 ± 2.4	52.1 ± 1.6	0.032
Birth weight (g)	3469 ± 723	3614 ± 644	3879 ± 225	0.121
Length (m)	1.66 ± 0.14	1.58 ± 0.16	1.58 ± 0.18	0.792
Weight (kg)	109.6 ± 31.5	90.1 ± 27.8	91.5 ± 29.3	0.859
BMI SDS	6.3 ± 0.9	5.5 ± 1.6	5.8 ± 1.5	0.038
P-Glucose (mmol/l)	4.5 ± 0.7	4.3 ± 0.6	4.4 ± 0.9	0.362
fS-Insulin (mmol/l)	117.7 ± 71.4	115.4 ± 74.6	92.6 ± 44.3	0.713
Triglycerides (mmol/l)	0.82 ± 0.80	0.81 ± 0.96	0.57 ± 0.53	0.882
Cholesterol (mmol/l)	3.73 ± 0.86	3.83 ± 0.86	3.43 ± 0.53	0.683

Data are means and SD divided into genotype groups. P indicates p-values adjusted for

significant covariates.

## Discussion

The TMEM18 gene has a remarkably long evolutionary history as it is found in most of the eukaryotic genomes we have looked at. It has therefore been present for at least 1500 MYA since the divergence of animals and plants/*T. pseudonana *[38]. Thus, it is one of the most ancient genes implicated in human obesity, such as FTO [39] and MC4R [40], that we are aware of. Most of the main genes involved in body weight regulation appeared after the radiation of vertebrates and very few are present beyond the animal kingdom. The amino acid sequences are highly conserved (Figures 2 and 3) with relatively small differences between vertebrates and other animals. Another very interesting feature (of curiosity, shared by the FTO gene), is that the gene is found in a single copy with no close relative in the genomes where it is present, despite that some genomes have undergone recent whole genome duplications such as *A. thaliana *(38 MYA) [41]. Surprisingly, we found two separate lineages where the TMEM18 gene is absent: fungi (yeasts, molds and mushrooms) and *pseudoocoelomata *(*C. elegans*). Both contain well analyzed genomes of high quality, suggesting that this gene has been lost at least two times on separate occasions in evolution and that the gene is thus not essential for life in all eukaryotes. The TMEM18 protein is predicted to have three transmembrane helices, a relatively uncommon property among human membrane proteins, as fewer than 5% of them share this feature [42]. The membrane topology is mainly found among proteins with poorly characterized functions that do not have close relatives and are, therefore, sole representatives of their protein family in the human proteome. The TMEM18 protein has a nuclear localization signal that targets the protein for transportation to the nucleus, which also has been confirmed experimentally [10]. Moreover, it contains a coiled-coil domain (Figure 1) that is common among DNA or RNA binding proteins, such a transcription factors, e.g. c-Fos [43]. However, transcription factors are generally not membrane-bound proteins, but at least one exception exists in the gene named Myelin gene regulatory factor (MRF) that is predicted to have a transmembrane helix and plays a central regulatory role in myelination of the CNS [44]. We found that there are only seven proteins, unrelated to each other, in the human proteome that contain three transmembrane helices and one coiled-coil domain, according to our queries in UniProt. Thus, the molecular structure of the TMEM18 protein is very uncommon in terms of other proteins in the human genome and surely very much different compared to other proteins involved in obesity, such as the enzyme FTO and the G protein-coupled melanocortin receptor, MC4R.

Other interesting features of the TMEM18 protein are the evolutionary conserved potential O-β-GlcNAc glycosylation and the phosphorylation sites, which are targets for the important posttranslational regulation of many proteins' functions and activities. Noteworthy, these sites are found in parts of the proteins that are supposed to face the inside of the nuclear envelope, i.e., the N-terminal and second loop (Figure 1) which, together with the beginning of the first and second transmembrane helices, are the most evolutionary conserved parts of the TMEM18 protein. This suggests that these parts of the protein constitute a potential active site, binding site or another important functional structure of the protein.

The TMEM18 gene is expressed in all the tissues we analyzed in the rat and mouse. Moreover, microarray data on TMEM18 from the fly (*D. melanogaster*), found in the FlyAtlas database, (Figure 4) correspond well to the mammalian expression data and confirm the widespread expression with the abundant presence in the brain, although higher in several other tissues. Thus, the expression pattern is in agreement with the evolutionary conservation of the protein sequence. We performed a comprehensive immunohistochemical analysis as well as *in situ *hybridization (Additional file 3). The immunohistochemical results showed that the TMEM18 protein is expressed throughout the mouse brain, which agrees with the real-time PCR expression profiles (Figure 4) and the *in situ *hybridization (See Additional file 3). We performed co-localization with specific neuronal marker and assessed the extent of the expression in five major brain areas. We found that TMEM18 is expressed in most cells (71%), with a somewhat higher, but significant (p = 0.011; t-test), presence in neurons (73%) than in non-neurons (64%). The outcome is consistent for different brain areas studied (Figure 5), while the hypothalamus is the only region that has a relatively lower percentage of expression in neurons. The TMEM18 gene was presented as a hypothalamic gene by the earlier study reporting the GWA to obesity [5]. We did not however see any evidence of significant enrichment in the TMEM18 expression level in the hypothalamus compared to other central regions, which is clear for, e.g., the aforementioned FTO gene and most neuroendocrine genes involved in body weight regulation. Thus, our results show that TMEM18 is likely to have a function in multiple tissues rather than being a solely hypothalamic gene. Nonetheless, the real-time PCR and immunohistochemical analyses confirmed that the TMEM18 is expressed abundantly in the hypothalamus and brainstem, regions that play a crucial role in the regulation of energy homeostasis: the hypothalamus, as the area that controls primarily feeding for calories and contains also components of the feeding reward system, and the brainstem that serves as the "relay station" for the gut-CNS signal cascade. Changes in energy needs of the organism, body weight as well as the rewarding value of food affect expression of genes known to participate in the regulation of consumption and body weight. Therefore, we studied the potential regulation of the TMEM18 gene expression in three different animal models that explore classical feeding paradigms. Feeding-related gene expression profile has previously been shown to be significantly changed in these tissues. For example, palatability affected hypothalamic expression of melanocortin receptor-4 (MC4R) and kappa (KOR) and mu (MOR) opioid receptors; an increased body weight influenced proopiomelanocortin (POMC), melanin concentrating hormone (MCH) and dynorphin (DYN) [45] while restriction of energy intake through deprivation upregulated expression of the obesity-associated gene FTO. Somewhat surprisingly, the TMEM18's expression level in the hypothalamus and brainstem remained the same in all models utilized herein (Figure 6). In fact, it shows a more stable expression (GeNorm expression stability value; M = 0.493) than the commonly used housekeeping genes: Tubulin beta (M = 0.575) in the brainstem samples from the deprivation and increased body weight models (Experiment 1 and 3) and Cyclophilin (M = 0.505, TMEM18 M = 0.458) in the hypothalamus samples from the Sucrose vs Intralipid model (Experiment 2). The average expression stability value is 0.469, which is remarkable as it parallels housekeeping genes in these models. Still, TMEM18 would not be considered a true housekeeping gene as it is not found in all cells. It is however clear that TMEM18 is differently regulated compared to the classical neuropeptides and receptors, whose mRNA levels are altered in these models. It is interesting in this context that TMEM18 contains several potential phosphorylation and glycosylation sites (Figure 1) as both these processes are common regulatory pathways for nuclear proteins. It is also possible that this gene is so prevalent in central neurons (73% of them express it) that feeding-related changes in TMEM18 expression might have affected only a small subpopulation of TMEM18 cells and they were not detectable with the real-time PCR analysis of a large region, such as the hypothalamus or brainstem.

Our human genetic study showed a strong significance for the association of two SNPs (Table 1) located upstream of TMEM18 with childhood obesity in a Swedish cohort of obese children and a clear trend for an increase in BMI SDS for homozygote carriers of the major allele. Further, the estimations of the odds-ratios suggest that there are significantly higher risks for carriers of the major allele of both SNPs to develop obesity (Table 1). The odds-ratio for rs6548238 (OR = 1.450) is in the same magnitude as the calculations made by Renström and colleagues (OR = 1.100) for a cohort of 4923 Swedish adults [7]. Moreover, the odds-ratios for the locus near TMEM18 are also as high as reported for the FTO locus (OR = 1.593) for the same cohort [35]. This strengthens previous observations that this region is as strongly associated with obesity as FTO. We do not find however any significant association for the variations with any of the investigated obesity-related traits, many relevant for the detection of a diabetic phenotype, while there is a trend for a decrease in birth weight and length for homozygote carriers of the major alleles (Table 2). In conclusion, the results show that a region near TMEM18 is significantly contributing to obesity in severely obese children, a group that arguably has the highest genetic component and greatest need for healthcare among different population subgroups of obese individuals.

## Conclusions

In conclusion, we show that common variations nearby the TMEM18 gene are associated not only to adult obesity but also obesity in severely obese children in a similar magnitude as FTO, the first gene associated with the common form of obesity. TMEM18 has a remarkably long evolutionary history that spans at least 1500 MY, longer than most other genes implicated in body weight regulation that we are aware of. The gene is widely distributed in the brain, found in the majority of all cells in the brain with slight, yet significant, enrichment in neurons compared to non-neurons. Expression of the gene does not seem to be affected in classical homeostatic models where several known regulators of appetite and reward are regulated. The protein has highly evolutionary conserved structural properties that induce the targeting to the nucleus of the cell, which may clarify why this newly identified gene has different expression and regulation patterns compared to the known neuroendocrine factors thus far associated with human obesity.

## Competing interests

The authors declare that they have no competing interests.

## Authors' contributions

MSA designed, conceived and performed the bioinformatical and evolutionary analysis and drafted the manuscript. JAJ carried out the genetic studies and drafted the manuscript. JHAS did the immunohistochemical and in situ hybdridization experiments. PKO designed, performed and analyzed the feeding experiments and participated in the writing of the draft. JC and JA designed, performed and analyzed the expression studies and contributed to the discussion of the manuscript. ASL designed and conceived the feeding experiments. RF, MC and HBS conceived the study, and participated in its design and the writing of the draft. All authors read and approved the final manuscript.

## Pre-publication history

The pre-publication history for this paper can be accessed here:

http://www.biomedcentral.com/1471-2350/11/58/prepub

## Supplementary Material

Additional file 1 TMEM18 specific primer sequences for the rat and mouse used for the real-time PCR analysis. Click here for file

Additional file 2 Protein sequences together with accession numbers for the TMEM18 proteins received in the mining and used in the subsequent analysis. Click here for file

Additional file 3 The figure shows *in situ *hybridization with the TMEM18 probe (red) and DAPI (blue) as a cellular marker in three regions of the mouse brain: **A**. Hypothalamus, **B**. Cortex and **C**. Amygdala. Click here for file
