# Supplementary material for: The obesity gene, TMEM18, is of ancient origin, found in majority of neuronal cells in all major brain regions and associated with obesity in severely obese children
Source: BMC Med Genet. 2010 Apr 9;11:58. doi: 10.1186/1471-2350-11-58 (PMC2858727; doi:10.1186/1471-2350-11-58)
Supplement: Additional file 1 — TMEM18 specific primer sequences for the rat and mouse used for the real-time PCR analysis. [file 1471-2350-11-58-S1.DOC]

Real-Time PCR primers

| **Mouse:** |  |
| --- | --- |
| ***Sense:*** | GCTCTGTGAATGGCTTATCTG |
| ***Antisense:*** | CTCTGTCACCTCAAATCTCTAAAG |
| **Rat:** |  |
| ***Sense:*** | TGTGATTATGTGGGTACGGAAGAC |
| ***Antisense:*** | CAACCAAGTCAGAGGACAAACAC |
