# Supplementary figures and images for: The obesity gene, TMEM18, is of ancient origin, found in majority of neuronal cells in all major brain regions and associated with obesity in severely obese children
Source: BMC Med Genet. 2010 Apr 9;11:58. doi: 10.1186/1471-2350-11-58 (PMC2858727; doi:10.1186/1471-2350-11-58)

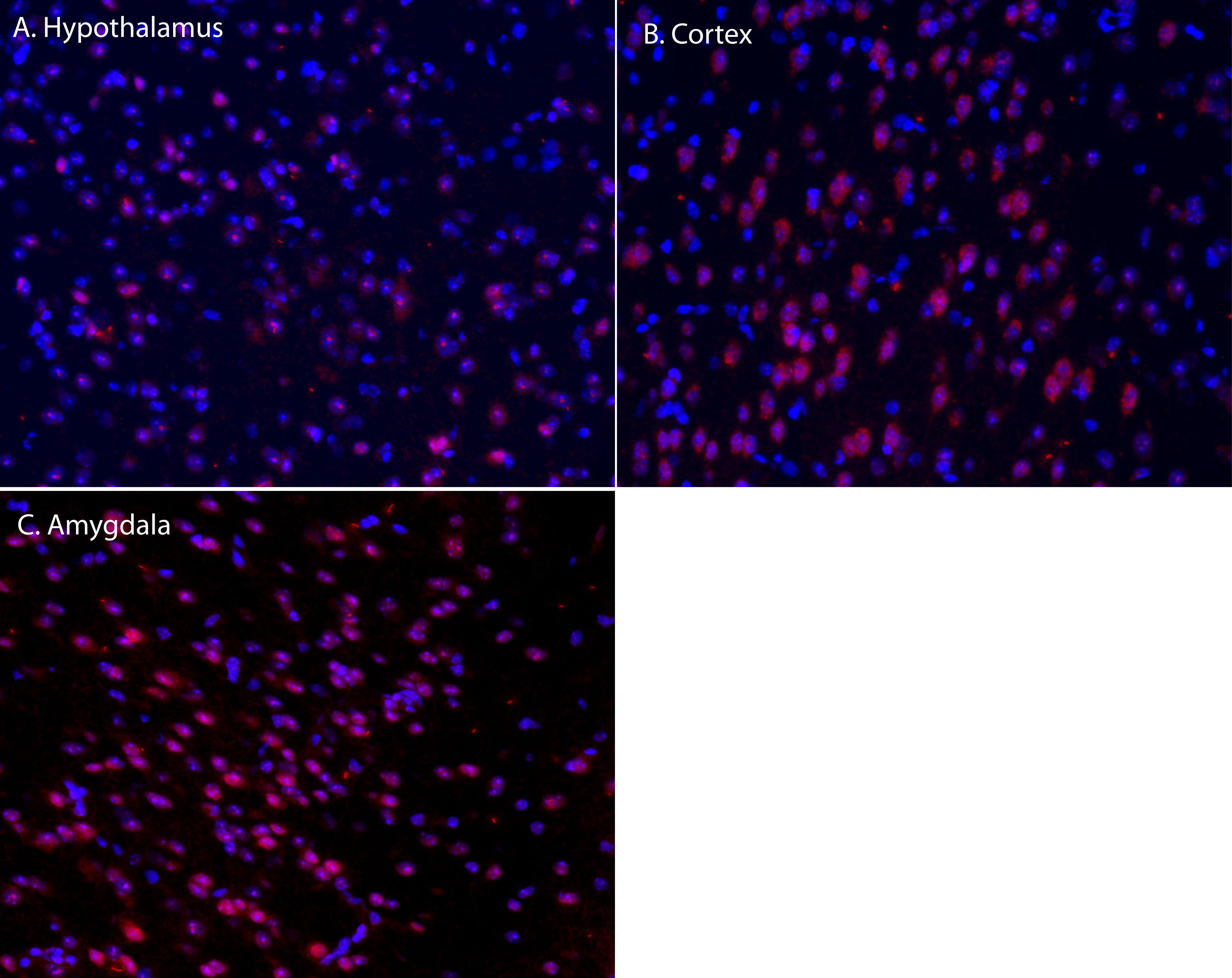

Supplement: Additional file 3 — The figure shows in situ hybridization with the TMEM18 probe (red) and DAPI (blue) as a cellular marker in three regions of the mouse brain: A. Hypothalamus, B. Cortex and C. Amygdala. [file 1471-2350-11-58-S3.JPEG]
